# Fatal injection of ranitidine: a case report

**DOI:** 10.1186/1752-1947-2-232

**Published:** 2008-07-17

**Authors:** Antonio Oliva, Sara Partemi, Vincenzo Arena, Fabio De Giorgio, Catia Colecchi, Nadia Fucci, Vincenzo L Pascali

**Affiliations:** 1 Institute of Legal Medicine, Catholic University, School of Medicine, Rome, Italy; 2 Institute of Pathology, Catholic University, School of Medicine, Rome, Italy; 3 Forensic Toxicology Laboratories, Catholic University, School of Medicine, Rome, Italy

## Abstract

**Introduction:**

Ranitidine hydrochloride (Zantac^®^), a histamine-2-receptor antagonist, is a widely used medication with an excellent safety record. Anaphylactic reaction to ranitidine is an extremely rare event and a related death has never been described in the literature.

**Case presentation:**

We present the clinical history, histological and toxicological data of a 51-year-old man with negative anamnesis for allergic events, who died suddenly after the intravenous administration of one phial of Zantac^® ^50 mg prescribed as a routine post-surgical prophylaxis for stress ulcer.

**Conclusion:**

Although the incidence of anaphylactic reactions related to ranitidine is low, caution needs to be exercised on administration of this drug. In addition, further study is needed to define strategies for the prevention of adverse drug reactions in hospitalized patients.

## Introduction

Ranitidine hydrochloride (Zantac^®^) is a histamine-2-receptor antagonist (H2RA) medication used in peptic ulcer disease therapy, acute stress ulcers, gastroesophageal reflux and related disorders (indications and dosages are summarized in Tables 1 and 2). This medication is often used intravenously in the operating room and during recovery in surgical departments or intensive care units, and orally in medical departments [1]. Ranitidine has an excellent safety record [2,3] and we found no reports of fatalities related to this drug in the literature, although the incidence of anaphylactic reaction to H2RAs and proton pump inhibitors together has been reported as 0.3% to 0.7% (see [4]). Several other adverse events are reported in clinical trials or in the routine management of patients treated with ranitidine [5]. Central nervous system symptoms such as malaise, dizziness, somnolence, insomnia and vertigo have been reported. Rare events of reversible mental confusion, agitation, depression and hallucinations have also been described, predominantly in severely ill elderly patients. Effects on the cardiovascular system have included rare cases of arrhythmias such as tachycardia, bradycardia, atrioventricular block and premature ventricular beats [6]. There have been occasional reports of hepatocellular, cholestatic or mixed hepatitis, with or without jaundice. These events are usually reversible, but in rare circumstances death has occurred. Cases of agranulocytosis, pancytopenia, sometimes with marrow hypoplasia, and aplastic anemia, and exceedingly rare events of acquired immune hemolytic anemia have been reported. A large epidemiological study suggested an increased risk of developing pneumonia in current users of H2RAs compared with patients who had stopped H2RA treatment. However, a causal relationship between the use of H2RAs and pneumonia has not been established.

**Table 1:** Ranitidine: indications and adult oral dosages

Indications	Dosages
Active duodenal ulcer	150 mg or 10 ml of syrup
Maintenance of healing of duodenal ulcers	150 mg or 10 ml of syrup
Pathological hypersecretory conditions (such as Zollinger-Ellison syndrome)	50 mg or 10 ml of syrup
Benign gastric ulcer	50 mg or 10 ml of syrup
Maintenance of healing of gastric ulcers	150 mg or 10 ml of syrup
Gastroesophageal reflux disease	150 mg or 10 ml of syrup
Erosive esophagitis	150 mg or 10 ml of syrup
Maintenance of healing of erosive esophagitis	150 mg or 10 ml of syrup

**Table 2:** Ranitidine: indications and adult intramuscular and intravenous dosages

Indications	Dosages
Treatment and maintenance for duodenal ulcer, hypersecretory conditions, gastroesophageal reflux.	**Intramuscular: **50 mg q 6–8 hr **Intermittent intravenous injection or infusion: **50 mg q 6–8 hr, not to exceed 400 mg/day. **Continuous intravenous infusion: **6.25 mg/hr

## Case presentation

A 51-year-old man was admitted to the hospital for treatment of benign prostatic hyperplasia (BPH). The patient's anamnesis was negative for allergic events. Before hospitalization he was being treated with alfuzosin, which belongs to a group of medications known as alpha-1A-receptor antagonists used to treat the symptoms of enlarged prostate and BPH. On admission to the hospital alfuzosin treatment was suspended and the patient underwent transurethral resection of the prostate under epidural anesthesia, followed by post-surgical administration of antibiotics (modivid) and lactated Ringer's solution. Twenty-four hours after surgery, routine prophylaxis for stress ulcer (one phial of Zantac^® ^50 mg, intravenous, in normal saline solution) was prescribed. Within minutes of the injection of ranitidine, the patient developed a combination of wheezing, dyspnea and hypotension followed by loss of consciousness. Despite intensive resuscitation attempts, no cardiac activity reappeared and death was certified 30 minutes later. As the circumstances of death appeared suspicious to the treating emergency physician, a forensic investigation was initiated and the public prosecutor ordered a forensic necropsy.

**Figure 1:**
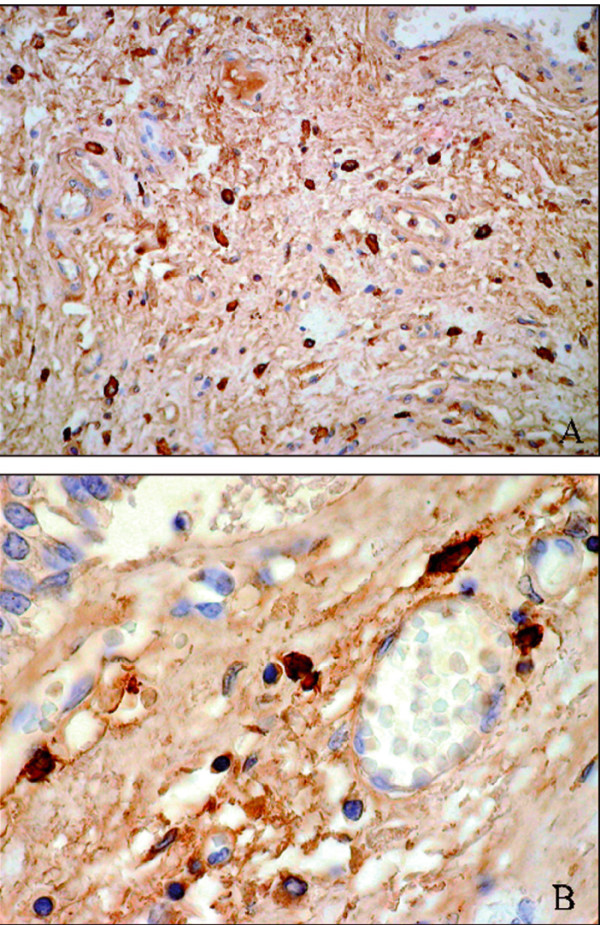
**Histological examination**. Immunohistochemical examinations (mouse anti – human monoclonal Mast Cell Tryptase (diluition 1:100; DAKO, Italy), demonstrated an increased number of mast cells in laryngeal submucosa (A) with perivascular localization (B).

The autopsy revealed pulmonary congestion with widespread upper airway edema, the presence of petechial hemorrhages and brain swelling with diffuse petechial hemorrhages. There was no evidence of recent myocardial infarction or other structural heart diseases. The rest of the organs were unremarkable. Histological sections confirmed the presence of widespread hypolaryngeal and pharyngeal mucosal and submucosal edema with inflammatory cells and an abundance of mast cells (Figure 1A and 1B). Testing for specific IgE antibodies and mast cell tryptase was not performed because of post-mortem degradation of the serum.

**Figure 2:**
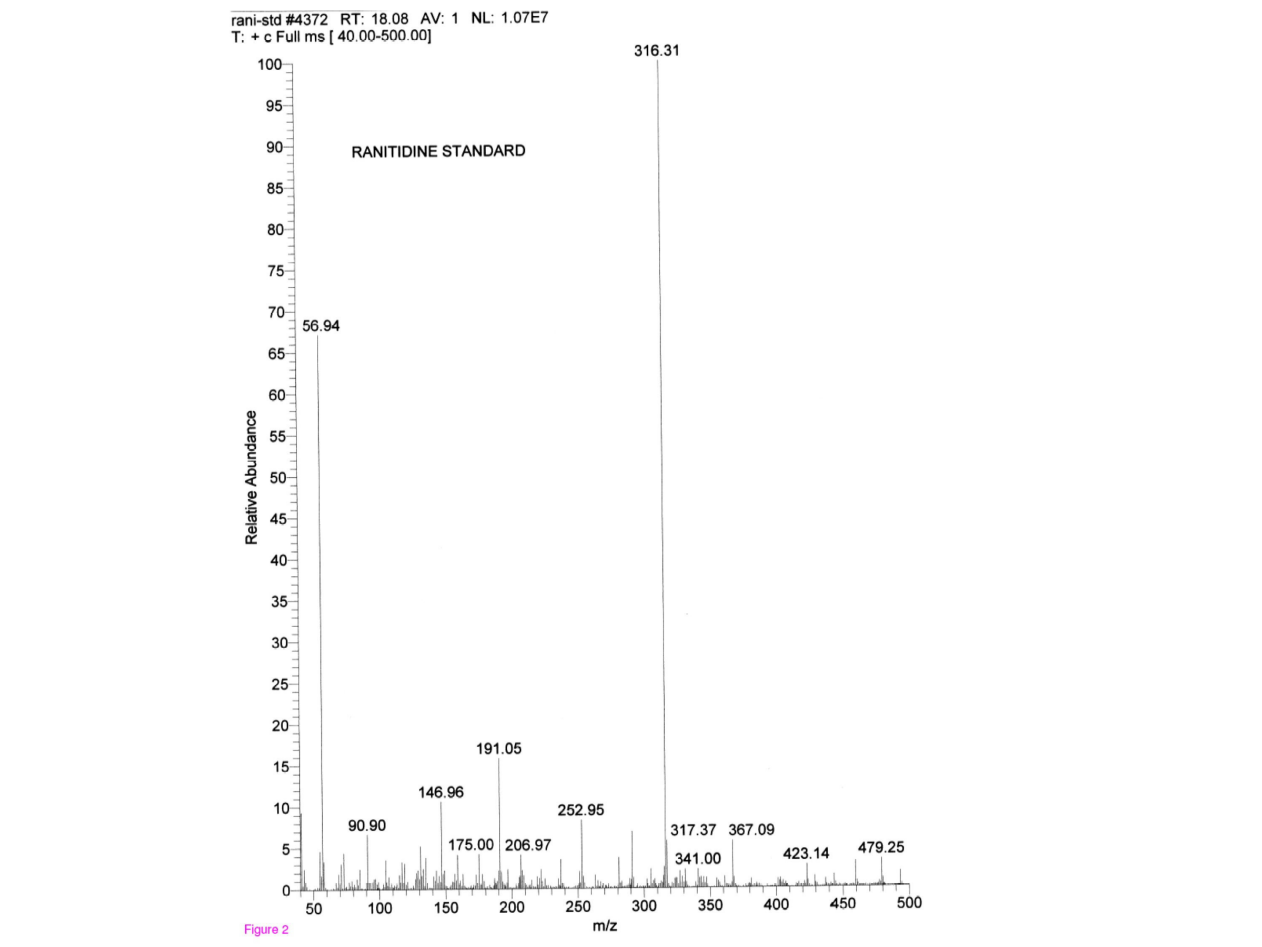
**Toxicological analysis**. Gas chromatography-mass spectrometry analysis shows the presence of ranitidine at the following concentrations: <10 ng/ml (limit of quantitation).

Toxicological analyses on blood performed using a gas chromatography-mass spectrometry technique revealed the presence of ranitidine at less than 10 ng/ml (limit of quantitation); see Figure 2. No other drugs were found. Death was attributed to anaphylactic shock due to an adverse reaction caused by intravenous injection of ranitidine, suggestive of a pathogenic mechanism of immediate-type hypersensitivity reaction type I, according to the Gell and Coombs Classification System.

## Discussion

Ranitidine, an H2RA which is commonly used to treat peptic ulcer and gastroesophageal reflux diseases, is associated with a low incidence of adverse reactions. Most reports were of obstetric patients [7,8], and a case of severe anaphylaxis to ranitidine in a patient with pancreatitis was also reported [9]. A review of the literature revealed no reported fatalities related to this drug.

We have presented the case of a 51-year-old man with negative anamnesis of allergic events, who was admitted to the hospital for treatment of BPH. During post-surgical recovery the patient received antibiotics (modivid), lactated Ringer's solution and, 24 hours after surgery, routine prophylaxis for stress ulcer (one phial of Zantac^® ^50 mg, intravenous, in normal saline solution) was added to the therapy. Within minutes of the injection of ranitidine clinical symptoms manifested in a combination of wheezing, dyspnea and hypotension followed by loss of consciousness and, despite intensive resuscitation attempts, no cardiac activity reappeared and death was certified 30 minutes later.

In accordance with the literature [10] both autopsy and histological investigations showed the most common post-mortem findings related to anaphylaxis, such as pulmonary congestion with upper airway edema, presence of petechial hemorrhages, brain swelling with diffuse petechial hemorrhages, the widespread presence of inflammatory cells and abundance of mast cells. Subsequent testing for specific IgE antibodies and mast cell tryptase was not performed because of post-mortem degradation of the serum. Despite these limitations, our results (clinical history, autopsy, histological and toxicological analyses) are highly suggestive of an anaphylactic reaction caused by ranitidine.

What lessons can learned from this case? It is hard to pick up a medical journal today without reading about some new medication, and how it promises to completely change the course of a disease or symptoms. The wonders of pharmacology are numerous but, as always, medications old or new are a double-edged sword. Much of the recent research on problems with medications has focused primarily on errors in medication use [11,12]. This is an important area of research, but adverse drug reactions (ADRs) that are not preventable, given our current state of knowledge, are a more common problem with a greater human burden. The question is whether the tracking of non-preventable drug-related injuries is important, especially when it is known that a specific drug can cause a specific reaction. It is important, for several reasons. Avoiding administration of the same medication to the patient in the future requires knowing and documenting that the patient had a previous allergy or sensitivity. When a patient develops an allergy or sensitivity, this information is often not recorded, resulting in patients receiving drugs to which they have known allergies or sensitivities: could this have happened in our case? Until the use of electronic medical records becomes ubiquitous [13], other initiatives must be undertaken to lower the incidence of ADRs. Health plans and pharmacy managers must work together to take effective steps to increase ADR monitoring and reporting and to proactively avoid ADRs through use of pharmacy management tools.

Another important and related issue is that hospitals have had strong incentives not to identify too many of these events [14]. Reporting large numbers of adverse events and any serious preventable event brings intense scrutiny from regulators and the public. Thus, most hospitals have relied on spontaneous reporting, which only identifies about 1 in 20 adverse reactions and leads to the perception that injuries from ADRs are less common than they really are [15].

For all of these reasons, areas of ongoing research need to be improved and directed toward diagnostic precision and accurate monitoring of ADRs, including further understanding of the immunochemistry of allergenic medications, improvement of the reproducibility and sensitivity of relevant IgE *in vitro *assays, and further validation of computer-assisted evaluation of adverse drug events. Moreover, the positive and negative predictive values for these diagnostic tests need to be better defined, whenever possible. At present, the primary diagnostic tool for properly assessing immunological drug reactions remains a meticulous and detailed history obtained by an astute, knowledgeable and motivated physician.

## Conclusion

We have described the only fatal reaction related to ranitidine in the literature to date. Reactions to this extensively used drug are very rare in clinical practice. However, this case suggests that, although the incidence of anaphylactic reactions related to ranitidine is low, caution needs to be exercised on administration of this drug. In addition, further study is needed to define strategies for the prevention of ADRs in hospitalized patients.

## Abbreviations

ADR: Adverse drug reaction; BPH: Benign prostatic hyperplasia; H2RA: Histamine-2-receptor antagonist.

## Competing interests

The authors declare that they have no competing interests.

## Consent

Written informed consent was obtained from the patient's next-of-kin for publication of this case report and accompanying images. A copy of the written consent is available for review by the Editor-in-Chief of this journal.

## Authors' contributions

AO and SP performed the autopsy examination and are responsible for the conception and design of the manuscript. VA performed the histological analysis. NF provided the toxicological results. FDG and CC performed the review of the literature. VLP is the supervisor of the manuscript. All the authors read and approved the final manuscript.

## Aknowledgments

This study has been supported by Fondi di Ateneo, Linea D1, Università Cattolica del Sacro Cuore.
